# Immunity and survival response of *Atta cephalotes* (Hymenoptera: Myrmicinae) workers to *Metarhizium anisopliae* infection: Potential role of their associated microbiota

**DOI:** 10.1371/journal.pone.0247545

**Published:** 2021-02-24

**Authors:** Sandra Milena Valencia-Giraldo, Andrea Niño-Castro, Andrea López-Peña, Danna Trejos-Vidal, Odair Correa-Bueno, James Montoya-Lerma

**Affiliations:** 1 Department of Biology, Universidad del Valle, Cali, Valle del Cauca, Colombia; 2 Center for the Study of Social Insects (CEIS), São Paulo State University (UNESP), Campus Rio Claro, Rio Claro, São Paulo, Brazil; USDA Agricultural Research Service, UNITED STATES

## Abstract

Leaf-cutting ants of the genera *Atta* and *Acromyrmex* are at constant risk of epizootics due to their dense living conditions and frequent social interactions between genetically related individuals. To help mitigate the risk of epizootics, these ants display individual and collective immune responses, including associations with symbiotic bacteria that can enhance their resistance to pathogenic infections. For example, *Acromyrmex* spp. harbor actinobacteria that control infection by *Escovopsis* in their fungal gardens. Although *Atta* spp. do not maintain symbiosis with protective actinobacteria, the evidence suggests that these insects are colonized by bacterial microbiota that may play a role in their defense against pathogens. The potential role of the bacterial microbiome of *Atta* workers in enhancing host immunity remains unexplored. We evaluated multiple parameters of the individual immunity of *Atta cephalotes* (Linnaeus, 1758) workers, including hemocyte count, encapsulation response, and the antimicrobial activity of the hemolymph in the presence or absence of bacterial microbiota. Experiments were performed on ants reared under standard conditions as well as on ants previously exposed to the entomopathogenic fungus *Metharrizium anisopliae*. Furthermore, the effects of the presence/absence of bacteria on the survival of workers exposed to *M*. *anisopliae* were evaluated. The bacterial microbiota associated with *A*. *cephalotes* workers does not modulate the number of hemocytes under control conditions or under conditions of exposure to the fungal pathogen. In addition, infection by *M*. *anisopliae*, but not microbiota, increases the encapsulation response. Similarly, the exposure of workers to this fungus led to increased hemolymph antimicrobial activity. Conversely, the removal of bacterial microbiota did not have a significant impact on the survival of workers with *M*. *anisopliae*. Our results suggest that the bacterial microbiota associated with the cuticle of *A*. *cephalotes* workers does not play a role as a modulator of innate immunity, either at baseline or after exposure to the entomopathogen *M*. *anisopliae*. Further, upon infection, workers rely on mechanisms of humoral immunity to respond to this threat. Overall, our findings indicate that the bacterial microbiota associated with *A*. *cephalotes* workers does not play a defensive role.

## Introduction

Leafcutter ants of the genera *Atta* and *Acromyrmex* are eusocial insects that live in colonies with a high density of genetically related individuals [1, 2]. These ants form a mutually beneficial symbiosis with the fungus *Leucoagaricus gongylophorus* [3–5]. In this association, ants provide the fungus with freshly cut leaves, and in return, the fungus grows gongylidia, specialized structures that comprise the main source of nutrition for the queen and the larvae [4, 6]. In principle, these living conditions should make ants prone to infectious disease outbreaks due to enhanced transmission between frequently interacting individuals susceptible to the same pathogens. Moreover, any infection that compromises the viability or productivity of the fungus garden will represent a risk for colony survival. Surprisingly, although a high abundance of pathogenic fungi nearby or within the nests in natural populations of leafcutter ants has been recorded, there is no evidence that those infections cause massive damage to the colonies [7–9]. In fact, evidence has revealed that fungus gardens can coexist with generalist fungi, including *Syncephalastrum racemosum* and *Trichoderma harzianum* [10].

On the other hand, fungus gardens are relatively vulnerable to infections by the specialized parasitic fungus *Escovopsis* [11, 12]. However, the mutualist overgrown by such fungi under optimal conditions, though unusual, occur frequently when the ants cannot sanitize the gardens and dispose of their waste properly [13–15]. This evidence suggests that the synergistic action of different immunity strategies of leafcutter ants is efficient in controlling microorganisms that pose a threat to ants or their mutualist fungus.

The immune defense of leafcutter ants consists of an individual immune system and social immunity. The innate immune system of these ants includes two mechanisms, humoral and cellular. The first involves antimicrobial peptides and the activation of enzymatic cascades that regulate hemolymph coagulation and melanization. At the same time, the cellular mechanism is composed of hemocytes that are responsible for phagocytosis and encapsulation of invasive organisms [16, 17]. Additionally, workers have a biomineral armor covering their exoskeleton that contributes to protecting them from the invasion of agents that penetrate the cuticle [18]. Furthermore, the antimicrobial secretions of several exocrine glands act as an external immune defense mechanism that constitutes a first barrier to pathogens and determines the microbial environment [19].

The collective immunity of leaf-cutting ants includes a division of labor in the colony [20, 21] and grooming or cleaning behaviors [1, 15] that are complemented with chemical defense mediated by metapleural gland secretions [19, 21]. In addition to these main levels of immunity, species of *Acromyrmex* show yet another association with actinobacteria symbionts of the genus *Pseudonocardia*, which are harbored on the cuticle of workers. The pivotal role of this association in immune defense has been demonstrated in the control of *Escovopsis* infections in the fungal garden [22–24]. In contrast, although *Atta* species maintain associations with black fungal species, it has not been established whether these fungi play a role in immune defense [25–28].

*Atta* species do not maintain a symbiosis with cuticular actinobacteria and appear to rely on fungus-grooming and weeding hygienic behaviors [29–31] as well as on antimicrobial compounds derived from different exocrine glands to control parasites that harm their symbiont gardens [32–34]. Although there are only a few studies that address the issue of microbiota composition in the genus *Atta* [35–38], it has been shown that they have an association with a bacterial community that includes a variety of actinobacteria in *A*. *sexdens* and *A*. *texana* [36, 38]. Furthermore, it has recently been suggested that similar to vertebrates, the insect gut is colonized by a bacterial microbiome, which plays an important role in nutrition and defense against pathogens [39–45]. Although the underlying mechanisms are not well understood, this protection appears to be mediated by toxins and antimicrobials. Other mechanisms involve modulation of immune responses by the host [46–48]. Given the superorganismal biology of leaf-cutting ants, fungus gardens are considered functionally equivalent to an external digestive system [49, 50]. Recent research has shown the presence of a fungus gardens microbiome with metabolic pathways involved in antimicrobial biosynthesis, which could suggest an interaction related to defense against pathogens [51]. The constant interaction between workers and the fungus makes the horizontal transfer of microbiota plausible, so the resident microbial communities on the workers may also have a role in defense.

On the other hand [52], showed that differences in the cuticular microbiota composition of the moth *Galleria mellonella* induced by the environment could significantly increase their susceptibility to the saprotrophic fungus *Conidiobolus coronatus*, suggesting that this bacterial community may play a role in the defense response against pathogens.

Since the defense reactions of insects, including *Acromyrmex* spp., may be affected by their associated bacterial microbiome, we evaluated the potential role of microbiota associated with *A*. *cephalotes* in their reactions to fungal infections. We hypothesized that the bacterial microbiota associated with *A*. *cephalotes* plays a potential role in the immune response of ant workers. We evaluated parameters of individual immunity, including hemocyte count, encapsulation response, and hemolymph antimicrobial activity in the presence and after removal of bacterial microbiota under basal conditions, as well as following fungal infection by the entomopathogenic fungus *Metharrizium anisopliae*. Likewise, we assessed the effect of the presence/absence of bacteria on the survival of workers challenged by this fungus.

## Materials and methods

### Nest selection and ant collection

Six independent colonies of *A*. *cephalotes* were chosen in a suburban area of Cali, Colombia (3°22’33.24” N, 76°32’0.24”W). As a selection criterion, the nest area was considered, which ranged between 35–50 m^2^. The existence of foraging tracks and the presence of workers carrying plant material to the nest and workers removing soil fragments were considered normal activity levels. Additionally, a massive exit of soldiers after disturbing the nest was verified. Between September 2018 and July 2019, 3000 major workers (cephalic width ≥ 2.1 mm) from each nest were collected by disturbing the nest entrances. These workers were maintained in plastic containers for at least 4 h before any experiment took place.

### Removal of bacterial microbiota

To establish the total abundance of bacterial microbiota, 100 individuals were collected from each nest. These ants were submerged in 2 ml of peptone broth (BD Bacto^TM^, Franklin Lakes, NJ, USA) in groups of five. The suspension was incubated for 3 h at 25°C. After this incubation, fivefold serial dilutions were seeded on plates of nutrient agar broth (BD-Bacto^TM^). The plates were incubated at 25°C for 72 h. Colonies were manually counted, and their abundance was reported as colony forming units (CFU) per ml.

To determine whether bacterial microbiota have an impact on the immune response of *A*. *cephalotes* workers, two treatments designated removal (R) and no removal (NR) were established. Each individual was held with forceps and completely submerged in a solution of 120 μg/ml gentamicin (GENFAR-Sanofi, Cali, Colombia) for 10 s to guarantee the contact of the entire ant with the solution, while workers from treatment NR were submerged in distilled water. To assess the efficiency of removal and before performing any assay, bacterial abundance was assessed before and after treatment with gentamicin or water, corroborating that gentamicin removed up to 97±1.7% of the bacterial microbiota.

After the treatments, the workers were maintained in groups of 10 individuals in Petri dishes for 24 h. During this time, they were fed *ad libitum* on portions of an agar-based diet as described by Bueno *et al*. [53].

To evaluate whether the bacterial microbiota has an impact on individual worker immunity under basal conditions and/or after challenge with *M*. *anisopliae*, we applied the R and NR treatments to the ants for each experiment. After 24 h, the workers were randomly divided into two equal groups: the first was left untreated, whereas workers of the second group were submerged individually for 10 s in a suspension of 1.5–3.5 x10^7^ conidia/ml of a strain of *M*. *anisopilae* derived from a commercial product (BIO-MA® Bioprotección, Manizales, Colombia) widely used in the control of insect pests.

### Hemocyte count and antimicrobial activity of hemolymph

To establish whether bacterial microbiota had an impact on hemocyte count either under basal conditions and/or after infection with *M*. *anisopliae*, we collected 240 major workers from each of the six nests selected for this study. Half of the individuals were selected to evaluate the effect of microbiota under basal conditions, and the remaining half were selected to assess the effect of the microbiota after infection with *M*. *anisopliae*. In both groups, workers were randomly assigned to either the R or NR treatments. After applying the treatments, we proceeded to extract hemolymph from the workers that were maintained under basal conditions. For the infection group, the hemolymph was extracted 24 h after challenge with *M*. *anisopliae*. In both cases, each worker was disinfected using 70% ethanol. Then, the head was separated from the thorax, and pressure was exerted on the subgenal region to collect the hemolymph in a micropipette. The hemolymph was then transferred to a microcentrifuge tube placed on ice.

The pooled hemolymph of five individuals, approx. 7 μl, was mixed with 3 μl of PBS. In total, we obtained eight samples for every experimental group. The samples were spread on microscope slides previously covered with poly-L-lysine at a concentration of 0.1 mg/ml (Sigma-Aldrich, Saint Louis, MO, USA). The slides were kept in a wet chamber at 4°C for at least 24 h to allow adherence of the hemocytes to the glass. Then, the hemocytes were stained with a DAPI solution (Santa Cruz Biotechnology, Dallas, TX, USA) at a concentration of 1 mg/ml and kept for 4 h in a dark chamber. The slides were washed thoroughly using PBS [54]. Hemocyte counts were performed using a Nikon MBA 92010 ECLIPSE Ni-U 90 epifluorescence microscope coupled to a Nikon MQA 16050 DS-Ri 1 U3 camera (Nikon, Melville, NY, USA). For each sample, images of 160 fields at 800x magnification were taken and analyzed using ImageJ software [55]. The results are plotted as the mean ± standard deviation.

To assess the hemolymph antimicrobial activity, 400 workers were captured from each colony. Collected workers underwent the R and NR treatments described above. After 24 h, ants from each treatment were assigned randomly to either the basal condition group or the infection group. The hemolymph of the ants in the basal condition group was extracted immediately, whereas ants from the infection group were treated with *M*. *anisopliae* spores, and hemolymph was extracted 24 h after infection. For both groups, we pooled the hemolymph extracted from five workers to form one sample, and those samples were kept on ice. Nutrient agar plates (nutrient broth BD Difco® and Bacto agar BD Difco® 1%, Franklin Lakes, NJ, USA) were inoculated with a strain of *Pseudomonas aeruginosa* isolated from the soil.

Immediately after inoculation, six discs of filter paper (Whatman® N°1, diameter 4 mm) were placed on each plate. On each plate, four discs were impregnated with hemolymph, and the remaining two were impregnated with PBS and gentamicin solution. The plates were incubated 24 h at 25°C. To calculate the diameter of the inhibition zone, we acquired images of each plate using a Nikon D5300 camera, and the images were analyzed using ImageJ software [55].

### Encapsulation response

Four nests were selected from each group to measure the encapsulation response under both basal and infection conditions, and 400 workers were collected and randomly assigned to R and NR treatments. After 24 h, half of the workers of each treatment were infected with *M*. *anisopliae* spores, and the other half were left untreated. Twenty-four hours after infection, the workers were briefly numbed on ice, and a 0.25 mm wide and 2.0 mm long nylon thread was implanted between the head and thorax. After 24 h, the implants were removed and placed on microscope slides in groups of 4, and we included a control implant that had not been introduced into ants on each slide. We captured images of each of the slides using a Nikon ECLIPSE Ci-L lens coupled to a Canon Ti3 camera using Helicon Focus software. The images were transformed to grayscale to calculate the mean gray-value parameter (MGV) for each implant using ImageJ software [55]. For the encapsulation assay, the result was reported as:

NormalizedMGV=1/MGVi‐MGVc

where MGVi represents the MGV measured for each implant, while MGVc is the MGV measured for the control implant.

### Survival assay after exposure to *Metarhizium anisopliae*

To assess the potential effect of the microbiota on the survival of workers challenged with *M*. *anisopliae*, 1000 individuals from six nests were assigned to the R and NR treatments. In addition, an uninfected control group was included (500 workers). The ants were kept in Petri dishes for 10 days as previously described. Cumulative survival was monitored every day. To verify that workers died due to *M*. *anisopliae* infection, corpses were disinfected with a 1% sodium hypochlorite solution and incubated in wet chambers until *M*. *anisopliae* hyphae were observed growing on their bodies.

### Data analyses

We performed statistical analyses using R software (R CoreTeam 2017). To assess whether removal of the microbiota had an impact on worker immunity, linear mixed models were used for each of the response variables: hemocyte count, diameter of the inhibition zone, MGV, and survival. The treatments (removal, no removal and control) were considered fixed effects, and the nest was considered a random effect. The hemocyte count data were log-transformed. To evaluate the significance of the fixed factor of each response variable, deviance analysis was performed using the CAR package ANOVA function [56]. To establish comparisons between groups, the Tukey test was performed using the multcomp package [57]. The significance level was set at α = 0.05 for all analyses. To assess the impact of the microbiota on worker survival after infection with *M*. *anisopliae*, we performed Cox regression using the survival package [58]. For this analysis, treatment (uninfected control, *M*. *anisopliae* infection in the presence of the microbiota and after the removal of the microbiota) was considered a predictor variable of the survival time of workers. Unless stated otherwise, data are presented as the mean ± standard deviation.

### Statement of ethical management

The collection of biological material for this study was covered by “Permiso marco de recolección de especímenes de especies silvestres de la diversidad biológica con fines de investigación científica no commercial” (Framework permit for the collection of specimens of wild species of biological diversity for noncommercial scientific research) issued by the National Environmental Licensing Authority (ANLA) to the Universidad del Valle through legal resolution N° 1070. The experimental procedures were reviewed and approved by the “Ethical Committee in fauna and flora research from the Faculty of Natural and Exact Sciences Universidad del Valle”. The favorable concept was stipulated in the concept document for research project No. 022–2016. All trials were performed after numbing the ants on ice to minimize affectation.

## Results

### Cellular immune response—hemocyte count

**Fig 1:**
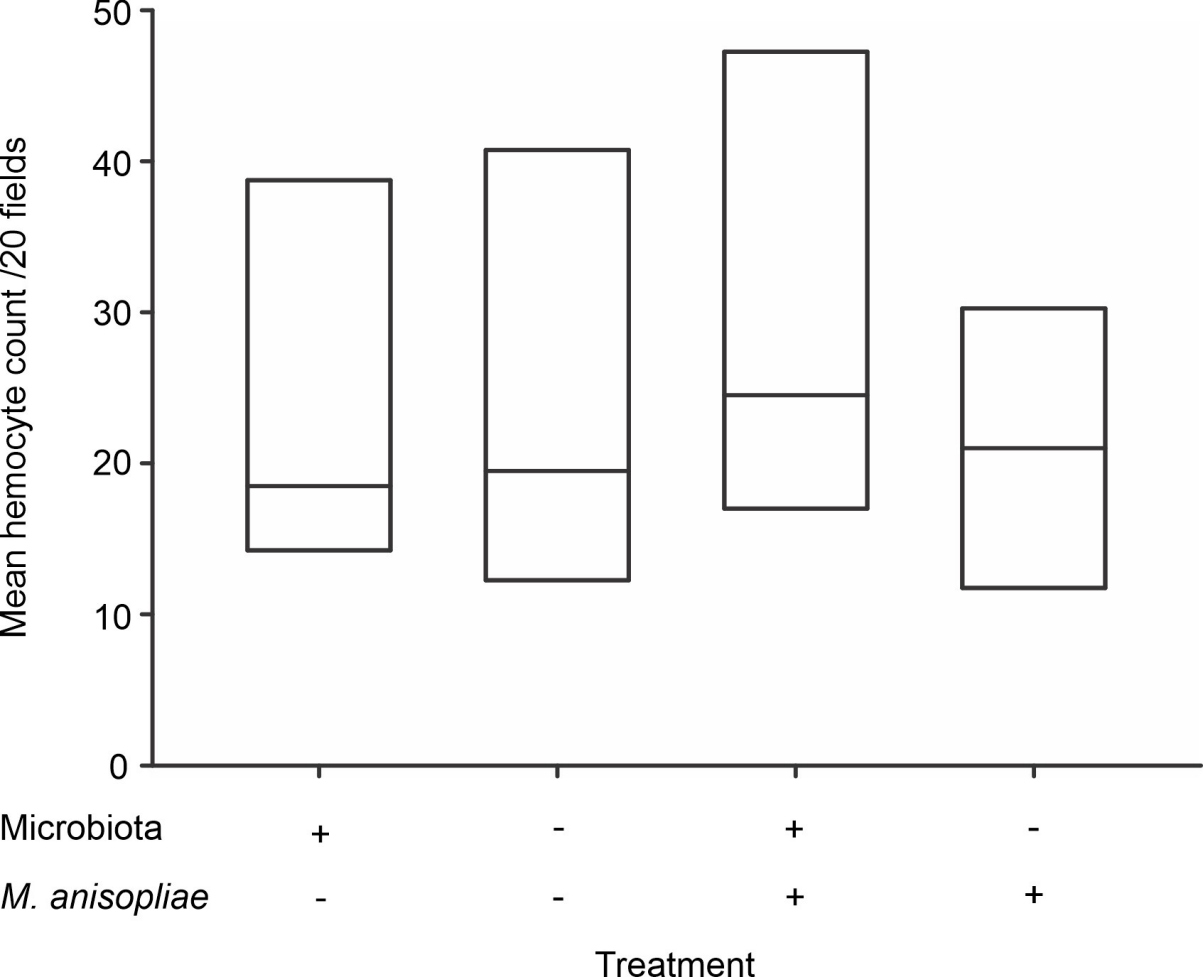
Hemocyte count in the hemolymph of *Atta cephalotes* workers in the presence of or after removal of the microbiota. Hemocyte count in *A*. *cephalotes* workers under basal conditions (left) and upon exposure (right) to *M*. *anisopliae* (N = 1440 workers from 6 nests).

The bacterial microbiota associated with workers did not modulate the number of circulating hemocytes in the hemolymph under basal conditions (χ^2^ = 0.67, p = 0.41) (Fig 1) or after exposure to the fungal pathogen *M*. *anisopliae* (χ^2^ = 2.18, p = 0.14). In addition, the results suggested that infection by *M*. *anisopliae* does not affect the hemocyte count; thus, no differences were observed between the uninfected control group and workers whose microbiota were not removed and were subsequently challenged by the fungus (Fig 1).

### Cellular immune response—encapsulation

**Fig 2:**
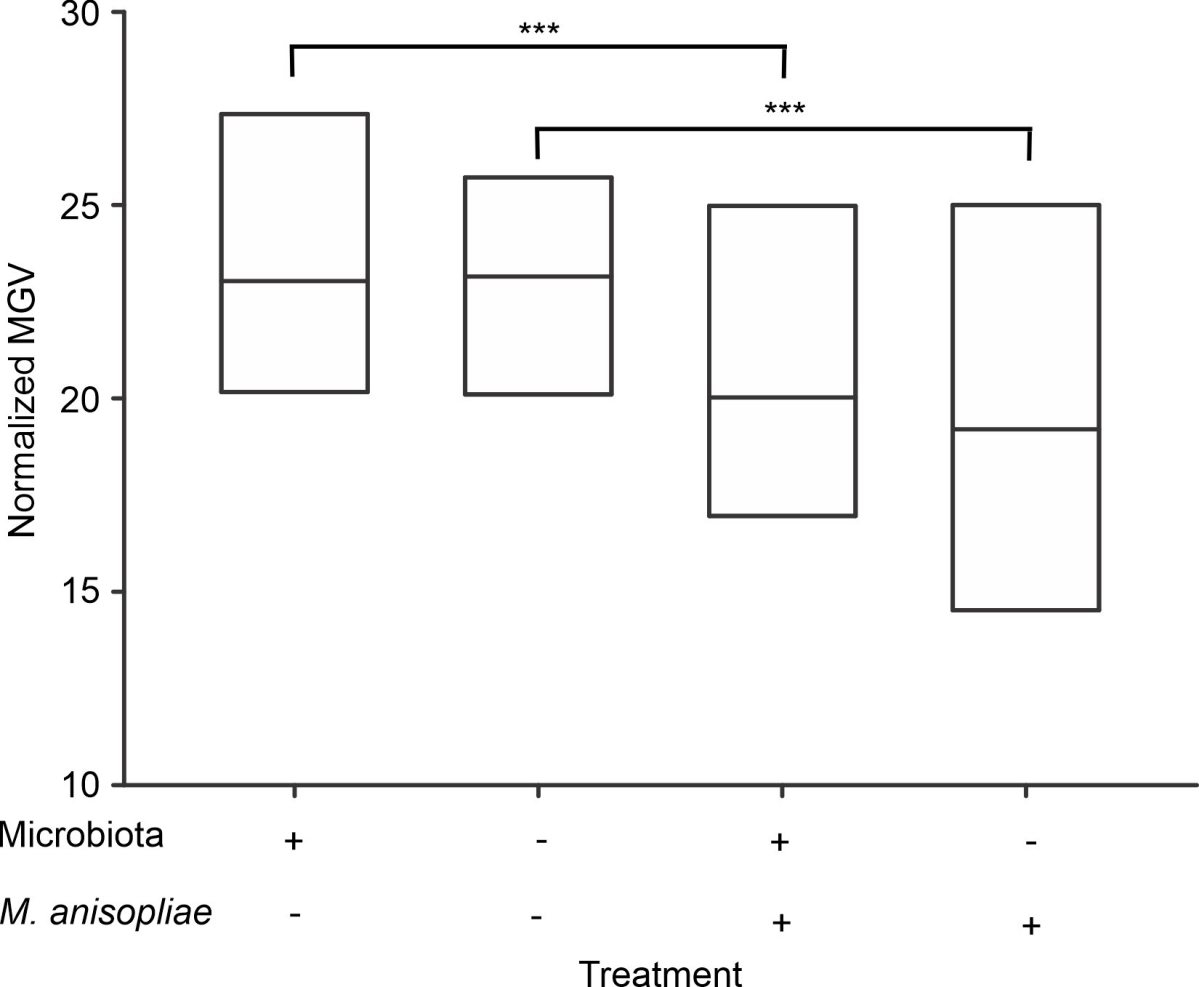
Encapsulation response of *Atta cephalotes* workers in the presence of or after removal of the microbiota. Normalized mean-gray value of implants extracted from workers subjected to the removal (left) and no removal (right) treatments. (N = 1600 workers from 4 nests) *** p <0.001.

The encapsulation assays showed that the treatments affected the encapsulation response (χ^2^ = 161.36, p<0.001). However, the comparisons between experimental groups indicated that infection with *M*. *anisopliae*, but not the presence of microbiota, modified the encapsulation response of *A*. *cephalotes* workers. Independent of the presence or absence of microbiota, infection by *M*. *anisopliae* caused a slight reduction (4.5%) in normalized MGV (Fig 2). This reduction was significant for ants in the presence of microbiota under basal conditions and after infection by *M*. *anisopliae* (p <0.001), as well as between ants in the absence of microbiota under basal conditions and after infection by *M*. *anisopliae* (p<0.001). However, we did not find significant differences between groups in the presence or absence of microbiota under basal conditions (p = 0.3615) or after infection by *M*. *anisopliae* (p = 0.8835) (Fig 2).

### Humoral response—antimicrobial activity of the hemolymph

**Fig 3:**
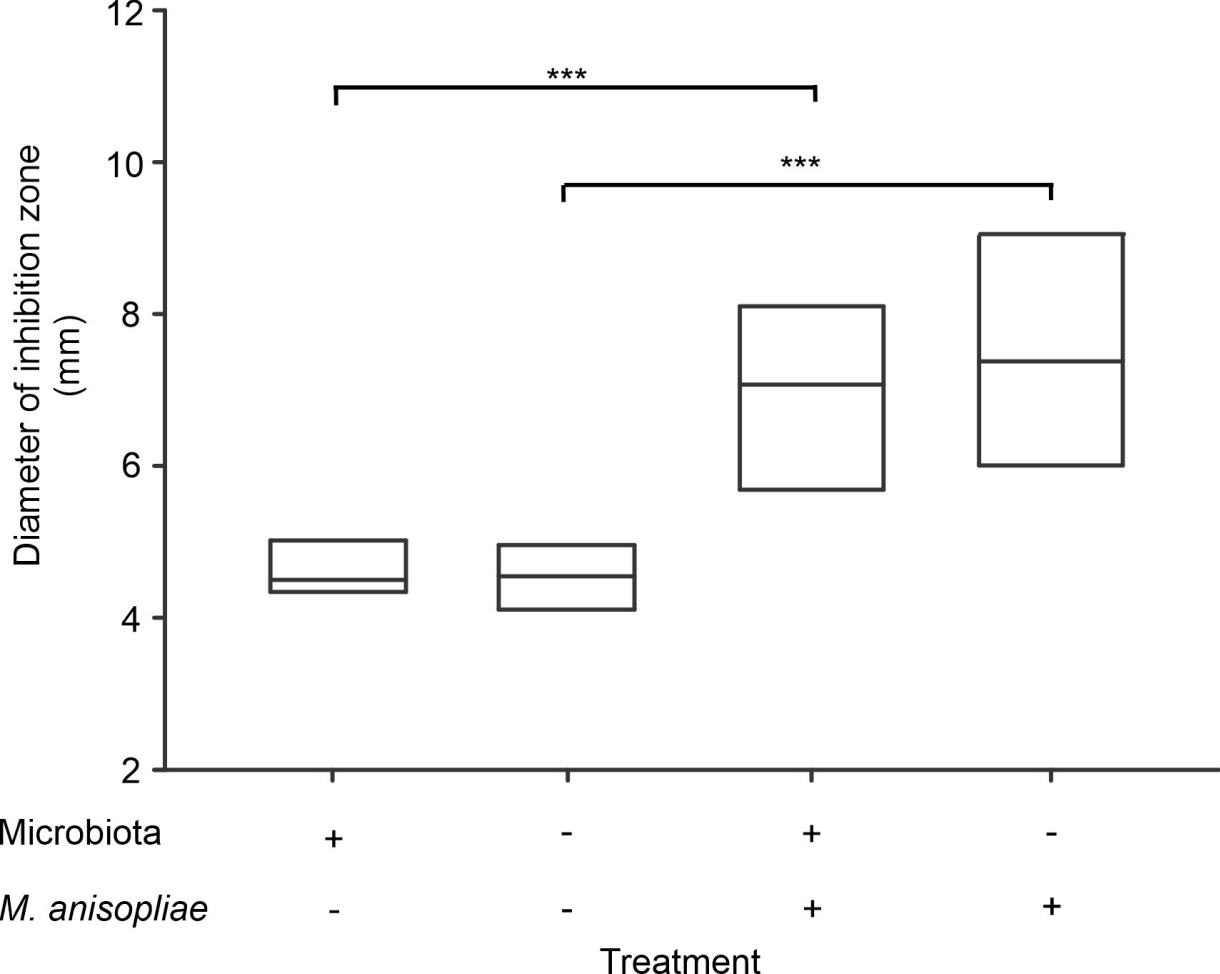
Antimicrobial activity of the hemolymph of *Atta cephalotes* workers in the presence of or after removal of the microbiota. Inhibition zone diameter was measured as an indicator of hemolymph antimicrobial activity in workers with intact microbiota and after removal under basal conditions and infection by *M*. *anisopliae* (N = 1200 workers from 4 nests *** p <0.01).

Similar to previous results, the treatments had an effect on hemolymph antimicrobial activity (χ^2^ = 179.6, p<0.001). However, infection by *M*. *anisopliae*, and not the removal of the microbiota, was a significant treatment. Independent of the presence or absence of microbiota, worker hemolymph showed low antimicrobial activity (Tukey test p = 0.98) under basal conditions. Likewise, there were no differences between hemolymph antimicrobial activity after infection among workers in the presence of and after removal of the microbiota (Tukey test p = 0.96). In contrast, the comparison between the hemolymph antimicrobial activity under basal conditions and after infection by *M*. *anisopliae* revealed that exposure of workers to this fungus led to an increase in the bactericidal activity of the hemolymph, showing an increase in the diameter of the inhibition zone. In line with this finding, significant differences in hemolymph antimicrobial activity were observed among workers under basal conditions and after infection in both the presence and absence of microbiota (Tukey test p<0.001) (Fig 3).

### Worker survival after infection by *M*. *anisopliae*

**Fig 4:**
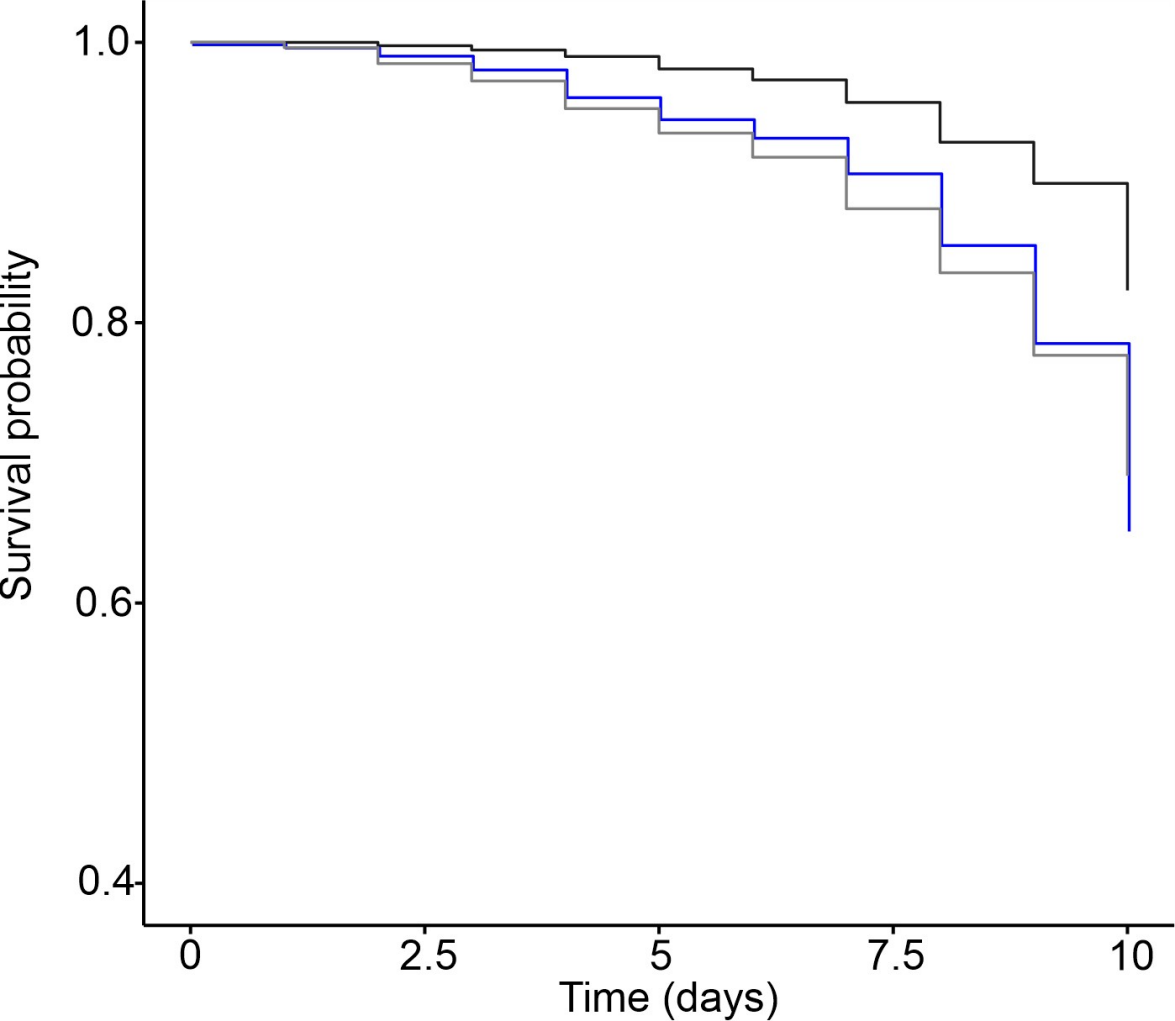
Survival probability of workers infected with *M*. *anisopliae* in the presence of and after removal of the microbiota. Survival probability of the uninfected workers (black line) and workers exposed to *M*. *anisopliae* in the presence of microbiota (blue line) and after removal of the microbiota (gray line). (N = 1500 workers from 5 nests).

Cox regression analysis showed that uninfected workers had a higher probability of survival than workers exposed to the entomopathogenic fungus *M*. *anisopliae* (Wald χ^2^ 28 *p <0*.*001)*. However, removal of the bacterial microbiota did not have a significant impact on the probability of survival of the infected workers (Wald χ^2^ 1.35 *p =* 0.24) (Fig 4).

## Discussion

Our results showed that the bacterial microbiota associated with *A*. *cephalotes* has no impact on individual ant worker immunity; its removal did not lead to any significant changes in hemocyte count, encapsulation response, and/or hemolymph antimicrobial activity, either under basal conditions or upon exposure to *M*. *anisopliae*. These results are consistent with those of the survival assay, where we did not observe any significant impact on worker survival following microbiota removal. In contrast, external workers of *Acromyrmex subterraneus*, which are naturally devoid of actinobacterial cover, are more resistant to entomopathogenic fungal infection than internal workers that exhibit an actinobacterial cover. This finding may suggest that under natural conditions, cuticular microbiota may exert immunomodulation on this species [59]. Nevertheless, these authors did not observe differences in the encapsulation rate of external workers covered by bacteria and internal workers without bacteria. Similar findings have been reported for *Ac*. *subterraneus* workers where removal of the visible actinobacterial cover did not influence the worker encapsulation rate [60]. This implies that bacterial coverage is not an exclusive modulator of the encapsulation rate and that it is likely worker age and/or their risk of exposure to pathogens shaped their response [59].

We only assessed the effect of bacterial microbiota on adult workers; therefore, we cannot rule out the role that interaction of the individual immune system and microbiota might play in immune defense at immature stages. This aspect is relevant since it has been reported that the composition of the *A*. *cephalotes* intestinal microbiota undergoes modifications during development. For instance, larval and pupal intestines contain *Pseudomonas* and *Enterobacter* bacteria, but adult intestines show a significant reduction in the abundance of these bacterial groups, suggesting that the constitution of the microbiota and, probably, its interaction with this host, are dynamic [38]. Additionally, an *Ac*. *subterraneus* actinobacterial cover appears to offer protection to the youngest workers until their immune system is fully developed [60]. This suggests that interactions with microorganisms may not be uniform throughout the worker lifespan.

Aside from the effect of microbiota on the immune response of workers, we assessed several parameters associated with the individual immune response of *A*. *cephalotes* workers under basal and infection conditions. Hemolymph exhibited virtually no antimicrobial activity under basal conditions, whereas microbicidal activity increased upon infection by *M*. *anisopliae*. This finding is in agreement with several studies on insects that have shown that antimicrobial peptide production is an inducible response triggered after the immune system recognizes threats [61–63]. Likewise, in *Ac*. *echinatior* workers, the expression of antimicrobial peptides is induced upon infection by entomompathogenic *M*. *anisopliae* [64]. Similarly, an increase in hemolymph antimicrobial activity after immune challenge has been reported in solitary insects such as *Galleria mellonella* [65], *Musca domestica* [66] and *Schistocerca gregaria* [67], as well as in social insects. For instance, it has been reported that *Bombus terrestris* [68] and *Apis mellifera* [69] bee workers challenged by bacterial-associated molecular patterns showed an increase in hemolymph antimicrobial activity compared to the control or uninfected group. Furthermore, *Camponotus fellah* worker hemolymph has no antimicrobial properties but it acquires such properties after a trauma [70].

The significant 4.5% reduction in encapsulation rate in the absence of any change in hemocyte count after 48 h of exposure to *M*. *anisopliae* is noteworthy. Considering that the encapsulation response is mainly mediated by hemocyte activity, our results suggest that in *A*. *cephalotes* workers, encapsulation results from humoral factors rather than cellular responses. Previous evidence revealed that upon infection with entomopathogenic fungi, secretion of antimicrobial peptides prevails over the cellular response [71, 72]. For instance, *Bombus terrestris* individuals challenged by bacterial lipopolysaccharide (LPS) showed enhanced hemolymph antimicrobial activity but did not exhibit changes in the hemocyte concentration [73]. Although we did not observe changes in the number of hemocytes circulating in the hemolymph, our results do not rule out the possibility that immune cells are recruited to the points where the invader penetrates the exoskeleton and protrudes into the hemocoel. This response has been reported in *Drosophila* larvae, where circulating hemocytes are rapidly recruited to the wounds, where they exert phagocytic activity [74]. Additional and similar functional assays were performed to determine why the hemocytes modified their functional profile, although they did not increase in number. This trait has been reported in *Aedes aegypti* hemocytes, which exhibit cell-specific transcriptome responses to infection [75].

A reduction in the encapsulation rate has previously been reported for *Ac*. *subterraneus subterraneus* and *Ac*. *equinatior* workers. In the latter, infection with *M*. *anisopliae* led to an ~40% reduction in worker encapsulation [64]. Our results showed a much less dramatic effect of *M*. *anisopliae* infection on the encapsulation rate, probably due to a shorter observation period. We evaluated the encapsulation rate at 48 h, while Baer et al. (2005) reported the strongest infection effect after 96 h. They did not observe any changes in the encapsulation response at 48 h. Regardless of the differences in the magnitude of the reduction, a reduced encapsulation response has been explained by the production of destruxins by *M*. *anisopliae* [76, 77]. These toxins prevent the formation of hemocyte aggregates and the prefenoloxidase reaction that together lead to a reduction in the encapsulation response [77]. Therefore, to determine whether *M*. *anisopliae* infection has a greater impact on the encapsulation response, as demonstrated in other leafcutter ant species, it would be necessary to assess this response at least 96 h after infection.

In agreement with our findings, the survival of workers challenged with *M*. *anisopliae* was not influenced by the presence/absence of bacteria, supporting the idea that the bacterial microbiota in *A*. *cephalotes* workers does not play a role in the defense against fungal infection. In line with this evidence, it has been proposed that in *Atta* species, which lack actinobacterial cover, the first line of defense against fungal invasion is the hygienic behaviors and chemical defense provided by metapleural gland activity [78]. In addition, *A*. *cephalotes* workers displayed an increase in the frequency of metapleural gland grooming during the first hour after challenge with the opportunistic pathogen *Penicillium* sp. [78]. Moreover, it has been shown that in *Atta colombica*, workers challenged, either by the entomopathogenic agent *M*. *anisopliae* or by the parasite *Escovopsis* sp., show a significant increase in grooming behavior frequency. Additionally, it has been found that *Atta* mandibular secretions can also minimize the action of toxic compounds and inhibit a wide range of microorganisms [79].

Our study evaluated, for the first time, the potential role of bacterial microbiota in the immune response of *A*. *cephalotes* and its effect on survival. Overall, our findings indicate that microbiota associated with *A*. *cephalotes* workers do not play a role in the defense against *M*. *anisopliae* and may support the hypothesis that in *Atta* species that are devoid of a visible actinobacterial coat, the behavioral and chemical arms of social immunity, including the secretions of the metapleural gland and fecal fluid, are the main mechanisms that restrict fungal infections.

## References

[1] Schmid-Hempel P. Parasites and social insects. Apidologie. 1995; 26(3): 255–271. 10.1051/apido:19950307

[2] Boomsma JJ, Schmid-Hempel P, Hughes WOH. Life histories and parasite pressure across the major groups of social insects. In: Fellowes M, Holloway G, Rolff J, editors. Insect Evolutionary Ecology. Royal Entomological Society. 2005; 139–175.

[3] Mueller UG, Gerardo NM, Aanen DK, Six DL, Schultz TR. The evolution of agriculture in insects. Annu Rev Ecol Evol Syst. 2005; 36: 563–595. 10.1146/annurev.ecolsys.36.102003.152626

[4] Chapela IH, Rehner SA, Schultz TR, Mueller UG. Evolutionary history of the symbiosis between fungus-growing ants and their fungi. Science. 1994; 266: 1691–1694. 10.1126/science.266.5191.1691 17775630

[5] Mueller UG, Schultz TR, Currie CR, Malloch D. The Origin of the Attine Ant-Fungus Mutualism. Q Rev Biol. 2001;76(2):169–197. 10.1086/393867 11409051

[6] Mueller UG, Scott JJ, Ishak HD, Cooper M, Rodrigues A. Monoculture of leafcutter ant gardens. PLoS One. 2010; 5(9): e12668 10.1371/journal.pone.0012668 20844760 PMC2937030

[7] Hughes WOH, Thomsen L, Eilenberg J, Boomsma JJ. Diversity of entomopathogenic fungi near leaf-cutting ant nests in a neotropical forest, with particular reference to *Metarhizium anisopliae* var. *anisopliae*. J. Invertebr. Pathol. 2004; 85(1):46–53. 10.1016/j.jip.2003.12.005 14992860

[8] Ribeiro MM, Amaral KD, Seide VE, Souza BM, Della Lucia T, Kasuya MC, et al Diversity of fungi associated with *Atta bisphaerica* (Hymenoptera: Formicidae): the activity *of Aspergillus ochraceus* and *Beauveria bassiana*. Psyche. 2012; 2012 10.1155/2012/389806

[9] Loreto RG, Hughes DP. Disease Dynamics in Ants: A Critical Review of the Ecological Relevance of Using Generalist Fungi to Study Infections in Insect Societies. Adv Genet. 2016; 94: 287–306. 10.1016/bs.adgen.2015.12.005 27131328

[10] Rodrigues A, Cable RN, Mueller UG, Bacci M, Pagnocca FC. Antagonistic interactions between garden yeasts and microfungal garden pathogens of leaf-cutting ants. Antonie van Leeuwenhoek. 2009; 96: 331–42. 10.1007/s10482-009-9350-7 19449210

[11] Currie CR, Mueller UG, Malloch D. The agricultural pathology of ant fungus gardens. Proceedings of the National Academy of Sciences. 1999 J; 96:7998–8002. 10.1073/pnas.96.14.7998 10393936 PMC22176

[12] Currie CR, Bot AN, Boomsma JJ. Experimental evidence of a tripartite mutualism: bacteria protect ant fungus gardens from specialized parasites. Oikos. 2003; 101: 91–102. 10.1034/j.1600-0706.2003.12036.x

[13] Bot ANM, Currie CR, Hart AG, Boomsma JJ. Waste management in leaf-cutting ants. Ethol Ecol Evol. 2001; 13(3):225–237. 10.1080/08927014.2001.9522772

[14] Heine D, Holmes NA, Worsley SF, Santos ACA, Innocent TM, Scherlach K, et al Chemical warfare between leafcutter ant symbionts and a co-evolved pathogen. Nat Commun. 2018; 9:1–11. 10.1038/s41467-017-02088-w 29880868 PMC5992151

[15] Reber A, Purcell J, Buechel SD, Buri P, Chapuisat M. The expression and impact of antifungal grooming in ants. J Evol Biol. 2011; 24(5):954–964. 10.1111/j.1420-9101.2011.02230.x 21306465

[16] Hoffmann JA. Innate immunity of insects. Curr Opin Immunol. 1995; 7(1):4–10. 10.1016/0952-7915(95)80022-0 7772280

[17] Tsakas S, Marmaras VJ. Insect immunity and its signalling: an overview. Invertebr. Surviv. J. 2010; 7(2): 228–238.

[18] Li H, Sun CY, Fang Y, Carlson CM, Xu H, Jesovnik A, et al Biomineral armor in leaf-cutter ants. Nat. Commun. 2020 10.1038/s41467-020-19566-3 33235196 PMC7686325

[19] Otti O, Tragust S, Feldhaar H. Unifying external and internal immune defences. Trends in ecology & evolution. 2014; 29: 625–634. 10.1016/j.tree.2014.09.002 25278329

[20] Ortiz-Reyes A, Giraldo-Jaramillo TM, Moreno-Herrera CX. Análisis molecular de las bacterias asociadas a los depósitos de desechos de *Atta cephalotes* (Hymenoptera: Formicidae). Rev Colomb Entomol. 2016; 42(2):162–70.

[21] Yek SH, Mueller UG. The metapleural gland of ants. Biol Rev. 2011; 86(4):774–791. 10.1111/j.1469-185X.2010.00170.x 21504532

[22] Cafaro MJ, Poulsen M, Little AEF, Price SL, Gerardo NM, Wong B, et al Specificity in the symbiotic association between fungus-growing ants and protective *Pseudonocardia* bacteria. Proc Royal Soc B. 2011; 278(1713):1814–1822. 10.1098/rspb.2010.2118 21106596 PMC3097832

[23] Poulsen M, Cafaro MJ, Erhardt DP, Little AEF, Gerardo NM, Tebbets B, et al Variation in *Pseudonocardia* antibiotic defence helps govern parasite-induced morbidity in *Acromyrmex* leaf-cutting ants. Environ Microbiol Rep. 2010; 2(4):534–40. 10.1111/j.1758-2229.2009.00098.x 22896766 PMC3418327

[24] Currie CR, Scott JA, Summerbell RC, Malloch D. Fungus growing ants use antibiotic producing bacteria to control garden parasites. Nature. 1999; 398:701–704. 10.1038/19519

[25] Pagnocca FC, Rodrigues A, Nagamoto NS, Bacci M. Yeasts and filamentous fungi carried by the gynes of leaf-cutting ants. Antonie Van Leeuwenhoek. 2008; 94:517–526. 10.1007/s10482-008-9268-5 18665453

[26] Guedes FLA, Attili-Angelis D, Pagnocca FC. Selective isolation of dematiaceous fungi from the workers of *Atta laevigata* (Formicidae: Attini). Folia Microbiol (Praha). 2012; 57:21–26. 10.1007/s12223-011-0081-6 22160859

[27] Duarte APM, Attili-Angelis D, Baron NC, Forti LC, Pagnocca FC. Leaf-cutting ants: An unexpected microenvironment holding human opportunistic black fungi. Antonie van Leeuwenhoek. 2014; 106:465–473. 10.1007/s10482-014-0215-3 24969946

[28] Duarte APM, Attili-Angelis D, Baron NC, Groenewald JZ, Crous PW, Pagnocca FC. Riding with the ants. Persoonia. 2017; 38:81–99. 10.3767/003158517X693417 29151628 PMC5645189

[29] Bass M, Cherrett JM. The role of leaf‐cutting ant workers (Hymenoptera: Formicidae) in fungus garden maintenance. Ecological Entomology. 1994; 19:215–220. 10.1111/j.1365-2311.1994.tb00412.x

[30] Currie CR, Stuart AE. Weeding and grooming of pathogens in agriculture by ants. Proceedings of the Royal Society of London. Series B: Biological Sciences. 2001; 268:1033–1039. 10.1098/rspb.2001.1605 11375087 PMC1088705

[31] Nilsson-Møller S, Poulsen M, Innocent TM. A visual guide for studying behavioral defenses to pathogen attacks in leaf-cutting ants. JoVE (Journal of Visualized Experiments). 2018; 140:e58420 10.3791/58420 30371666 PMC6235524

[32] Hölldobler B, Wilson EO. The ants. Harvard University Press; 1990 10.1007/BF01021020

[33] Ortius-Lechner D, Maile R, Morgan ED, Boomsma JJ. Metapleural gland secretion of the leaf-cutter ant Acromyrmex octospinosus: new compounds and their functional significance. Journal of Chemical Ecology. 2000; 26:1667–1683. 10.1023/A:1005543030518

[34] Fernandez-Marin H, Zimmerman JK, Nash DR, Boomsma JJ, Wcislo WT. Reduced biological control and enhanced chemical pest management in the evolution of fungus farming in ants. Proc Royal Soc B. 2009; 276(1665):2263–2269. 10.1098/rspb.2009.0184 19324734 PMC2677613

[35] Vieira AS, Ramalho MO, Martins C, Martins VG, Bueno OC. Microbial Communities in Different Tissues of *Atta sexdens rubropilosa* Leaf-cutting Ants. Curr Microbiol. 2017; 74:1216–1225. 10.1007/s00284-017-1307-x 28721658

[36] Ramalho M de O, Martins C, Morini MSC, Bueno OC. What can the bacterial community of *Atta sexdens* (Linnaeus, 1758) tell us about the habitats in which this ant species evolves?. Insects. 2020; 11(6): 1–20. 10.3390/insects11060332 32481532 PMC7349130

[37] Montoya QV, Meirelles LA, Chaverri P, Rodrigues A. Unraveling *Trichoderma* species in the attine ant environment: description of three new taxa. Antonie van Leeuwenhoek. 2016; 109:633–651. 10.1007/s10482-016-0666-9 26885975

[38] Zhukova M, Sapountzis P, Schiøtt M, Boomsma JJ. Diversity and transmission of gut bacteria in *Atta* and *Acromyrmex* leaf-cutting ants during development. Front Microbiol. 2017; 8:1–14. 10.3389/fmicb.2017.00001 29067008 PMC5641371

[39] Engel P, Moran NA. The gut microbiota of insects—diversity in structure and function. FEMS Microbiol Rev. 2013; 37(5): 699–735. 10.1111/1574-6976.12025 23692388

[40] Dillon RJ, Vennard CT, Buckling A, Charnley AK. Diversity of locust gut bacteria protects against pathogen invasion. Ecol Lett. 2005; 8(12):1291–1298. 10.1111/j.1461-0248.2005.00828.x

[41] Gurung K, Wertheim B, Salles JF. The microbiome of pest insects: it is not just bacteria. Entomol Exp Appl. 2019; 167(3):156–170. 10.1111/eea.12768

[42] Wei G, Lai Y, Wang G, Chen H, Li F, Wang S. Insect pathogenic fungus interacts with the gut microbiota to accelerate mosquito mortality. Proc Natl Acad Sci U S A. 2017; 114(23): 5994–5999. 10.1073/pnas.1703546114 28533370 PMC5468619

[43] Strand MR. The gut microbiota of mosquitoes: Diversity and function. In: Wikel S, Aksoy S, Dimopoulos G, editors. Arthropod vector: Controller of disease transmission. Academic Press; 2017 pp. 185–199.

[44] Lemaitre B, Miguel-Aliaga I. The Digestive Tract of *Drosophila melanogaster*. Annu Rev Genet. 2013; 47:377–404. 10.1146/annurev-genet-111212-133343 24016187

[45] Dillon RJ, Dillon VM. The gut bacteria of insects: nonpathogenic interactions. Annu Rev Entomol. 2004; 49:71–92. 10.1146/annurev.ento.49.061802.123416 14651457

[46] Cory JS. Evolution of host resistance to insect pathogens. Curr Opin Insect Sci. 2017; 21:54–59. 10.1016/j.cois.2017.04.008 28822489

[47] Van Arnam EB, Currie CR, Clardy J. Defense contracts: Molecular protection in insect-microbe symbioses. Chem Soc Rev. 2018; 47(5):1638–1651. 10.1039/c7cs00340d 28745342

[48] Oliver KM, Martinez AJ. How resident microbes modulate ecologically-important traits of insects. Curr Opin Insect Sci. 2014; 4:1–7. 10.1016/j.cois.2014.08.001 28043402

[49] Aylward FO, Burnum KE, Scott JJ, Suen G, Tringe SG, Adams SM, et al Metagenomic and metaproteomic insights into bacterial communities in leaf-cutter ant fungus gardens. The ISME journal. 2012; 9:1688–1701. 10.1038/ismej.2012.10 22378535 PMC3498920

[50] Somera AF, Lima AM, dos Santos-Neto ÁJ, Lanças FM, Bacci M. Leaf-cutter ant fungus gardens are biphasic mixed microbial bioreactors that convert plant biomass to polyols with biotechnological applications. Applied and environmental microbiology. 2015; 81:4525–4535. 10.1128/AEM.00046-15 25911490 PMC4475879

[51] Barcoto MO, Carlos-Shanley C, Fan H, Ferro M, Nagamoto NS, Bacci M, et al Fungus-growing insects host a distinctive microbiota apparently adapted to the fungiculture environment. Sci Rep. 2020; 10: 1–3. 10.1038/s41598-019-56847-4 32709946 PMC7381635

[52] Kazek M, Kaczmarek A, Wrońska AK, Boguś MI. Diet influences the bacterial and free fatty acid profiles of the cuticle of *Galleria mellonella* larvae. PLoS One. 2019; 14(2): e0211697 10.1371/journal.pone.0211697 30730940 PMC6366757

[53] Bueno OC, Hebling MJA, Silva OA, Matenhauer AMC. Effect of sesame (*Sesamum indicum* L) on nest development of *Atta sexdens rubropilosa* Forel (Hym., Formicidae). J Appl Entomol. 1995; 119(1–5): 341–343. 10.1111/j.1439-0418.1995.tb01297.x

[54] Baer B, Armitage SAO, Boomsma JJ. Sperm storage induces an immunity cost in ants. Nature. 2006; 441(7095):872–875. 10.1038/nature04698 16778889

[55] Rueden CT, Schindelin J, Hiner MC, DeZonia BE, Walter AE, Arena ET, et al ImageJ2: ImageJ for the next generation of scientific image data. BMC Bioinformatics. 2017; 18(1). 10.1186/s12859-017-1934-z 29187165 PMC5708080

[56] Fox J, Weisberg S. An {R} Companion to Applied Regression. Second Thousand Oaks {CA}: Sage; 2011.

[57] Hothorn T, Bretz F, Westfall P. Simultaneous Inference in General Parametric Models. Biom J. 2008; 50(3):346–363. 10.1002/bimj.200810425 18481363

[58] Therneau TM. A Package for Survival Analysis in R. R package version 3.2–3. 2020. Available from: https://CRAN.R-project.org/package=survival.

[59] Couceiro J da C, Marcelino WL, Amaral KD, Gandra LC, de Souza DJ, Della Lucia TMC. Effects of entomopathogenic fungi on the mortality and immune system of the leaf-cutting ant *Acromyrmex subterraneus subterraneus*. Entomol Exp Appl. 2016; 161(2):152–159. 10.1111/eea.12500

[60] De Souza DJ, Lenoir A, Kasuya MCM, Ribeiro MMR, Devers S, Couceiro J da C, et al Ectosymbionts and immunity in the leaf-cutting ant *Acromyrmex subterraneus subterraneus*. Brain Behav Immun. 2013; 28:182–187. 10.1016/j.bbi.2012.11.014 23207105

[61] Lemaitre B, Reichhart JM, Hoffmann JA. *Drosophila* host defense: Differential induction of antimicrobial peptide genes after infection by various classes of microorganisms. Proc Natl Acad Sci U S A. 1997;94(26):14614–14619. 10.1073/pnas.94.26.14614 9405661 PMC25070

[62] Gillespie JP, Kanost MR, Trenczek T. Biological mediators of insect immunity. Annu Rev Entomol. 1997; 42:611–643. 10.1146/annurev.ento.42.1.611 9017902

[63] Hoffmann JA, Hetru C. Insect defensins: inducible antibacterial peptides. Immunol Today. 1992; 13(10):411–415. 10.1016/0167-5699(92)90092-L 1418378

[64] Baer B, Krug A, Boomsma JJ, Hughes WOH. Examination of the immune responses of males and workers of the leaf-cutting ant *Acromyrmex echinatior* and the effect of infection. Insectes Soc. 2005; 52(3):298–303. 10.1007/s00040-005-0809-x

[65] Andrejko M, Siemińska A. The role of *Pseudomonas aeruginosa* alkaline protease in activation of the antimicrobial activity in *Galleria mellonella* larvae. Invertebrate Surviv J. 2016; 13:269–280. 10.25431/1824-307X/isj.v13i1.269–280

[66] Zhang G, Cowled C, Shi Z, Huang Z, Bishop-Lilly KA, Fang X, et al Comparative analysis of bat genomes provides insight into the evolution of flight and immunity. Science. 2013; 339(6118):456–460. 10.1126/science.1230835 23258410 PMC8782153

[67] Xia Y, Dean P, Judge AJ, Gillespie JP, Clarkson JM, Charnley AK. Acid phosphatases in the haemolymph of the desert locust, *Schistocerca gregaria*, infected with the entomopathogenic fungus *Metarhizium anisopliae*. J Insect Physiol. 2000; 46(9):1249–1257. 10.1016/S0022-1910(00)00045-7 10844143

[68] Moret Y, Schmid-Hempel P. Survival for immunity: The price of immune system activation for bumblebee workers. Science. 2000; 290(5494):1166–1168. 10.1126/science.290.5494.1166 11073456

[69] Riessberger-Gallé U, Hernández López J, Schuehly W, Crockett S, Krainer S, Crailsheim K. Immune responses of honeybees and their fitness costs as compared to bumblebees. Apidologie. 2015; 46(2):238–249. 10.1007/s13592-014-0318-x 26412907 PMC4579911

[70] De Souza DJ, Van Vlaenderen J, Moret Y, Lenoir A. Immune response affects ant trophallactic behaviour. J Insect Physiol. 2008; 54(5):828–832. 10.1016/j.jinsphys.2008.03.001 18430435

[71] Charnley AK, Collins SA. Entomopathogenic fungi and their role in pest control. In: Kubicek CP, Druzhinina IS, editors. The mycota: Environmental and microbial relationships. Second New York: Springer; 2007 pp. 159–188. 10.1007/978-3-540-71840-6_10

[72] Gillespie JP, Bailey AM, Cobb B, Vilcinskas A. Fungi as elicitors of insect immune responses. Arch Insect Biochem Physiol. 2000; 44:49–68. 10.1002/1520-6327(200006)44:2&lt;49::AID-ARCH1&gt;3.0.CO;2-F 10861866

[73] Moret Y, Schmid-Hempel P. Immune responses of bumblebee workers as a function of individual and colony age: Senescence versus plastic adjustment of the immune function. Oikos. 2009; 118:371–378. 10.1111/j.1600-0706.2008.17187.x

[74] Babcock DT, Brock AR, Fish GS, Wang Y, Perrin L, Krasnow MA, et al Circulating blood cells function as a surveillance system for damaged tissue in *Drosophila* larvae. Proc Natl Acad Sci U S A. 2008; 105(29):10017–10022. 10.1073/pnas.0709951105 18632567 PMC2474562

[75] Choi YJ, Fuchs JF, Mayhew GF, Yu HE, Christensen BM. Tissue-enriched expression profiles in *Aedes aegypti* identify hemocyte-specific transcriptome responses to infection. Insect Biochem Mol Biol. 2012; 42:729–738. 10.1016/j.ibmb.2012.06.005 22796331 PMC3438353

[76] Huxham IM, Lackie AM, McCorkindale NJ. Inhibitory effects of cyclodepsipeptides, destruxins, from the fungus *Metarhizium anisopliae*, on cellular immunity in insects. J Insect Physiol. 1989; 35(2):97–105. 10.1016/0022-1910(89)90042-5

[77] Cerenius L, Trörnqvist PO, Vey A, Johansson MW, Söderhäll K. The effect of the fungal toxin destruxin E on isolated crayfish haemocytes. J Insect Physiol. 1990; 36(10):785–789. 10.1016/0022-1910(90)90052-H

[78] Fernández-Marín H, Zimmerman JK, Rehner SA, Wcislo WT. Active use of the metapleural glands by ants in controlling fungal infection. Proc R Soc B Biol Sci. 2006; 273(1594):1689–1695. 10.1098/rspb.2006.3492 16769642 PMC1634922

[79] Samuels RI, Mattoso TC, Moreira DDO. Chemical warfare: Leaf-cutting ants defend themselves and their gardens against parasite attack by deploying antibiotic secreting bacteria. Commun Integr Biol. 2013; 6(2):e23095 10.4161/cib.23095 23795235 PMC3609840

